# A comparison of two tests for filarial antigenemia in areas in Sri Lanka and Indonesia with low-level persistence of lymphatic filariasis following mass drug administration

**DOI:** 10.1186/s13071-015-0979-y

**Published:** 2015-07-15

**Authors:** Thishan C. Yahathugoda, Taniawati Supali, Ramakrishna U. Rao, Yenny Djuardi, Difa Stefani, Femmy Pical, Peter U. Fischer, Melanie M. Lloyd, Prasad H. Premaratne, Mirani V. Weerasooriya, Gary J. Weil

**Affiliations:** Filariasis Research, Training and Service Unit, Department of Parasitology, Faculty of Medicine, University of Ruhuna, Galle, Sri Lanka; Department of Parasitology, Faculty of Medicine, University of Indonesia, Jakarta, Indonesia; Department of Internal Medicine, Infectious Diseases Division, Washington University School of Medicine, St. Louis, MO USA; Department of Para Clinical Sciences, Faculty of Medicine, General Sir John Kotelawala Defence University, Kandawala Estate, Ratmalana, Sri Lanka

**Keywords:** Lymphatic filariasis, *Wuchereria bancrofti*, Diagnosis, Antigenemia, Elimination, Mass drug administration, Sri Lanka, Indonesia

## Abstract

**Background:**

Filarial antigen tests are key tools for mapping the distribution of bancroftian filariasis and for detecting areas with persistent infections following mass drug administration (MDA). A recent study showed that the new Alere Filariasis Test Strip (FTS) has better analytical sensitivity than the BinaxNOW Filariasis card test (Card Test) for detecting circulating filarial antigen, and the FTS detected more positive results than the Card Test in a field study performed in a highly endemic area in Liberia.

**Methods:**

The present study compared the performance of the FTS and the Card Test in community surveys that were conducted in southern Sri Lanka and in Indonesia (Central Java) in areas with low-level persistence of LF following multiple rounds of MDA with diethylcarbamazine plus albendazole. The studies were performed in densely populated semi-urban areas where *Wuchereria bancrofti* is transmitted by *Culex quinquefasciatus*.

**Results:**

Antigenemia rates by FTS were 138 % higher in the Sri Lanka study (43/852 vs. 18/852) and 21 % higher in the Indonesia study (50/778 vs. 41/778) than antigenemia rates by Card Test. Antigenemia rates were significantly higher in males than in females and higher in adults than in children in both study sites. Although overall antigenemia rates and test scores were significantly higher by FTS than by Card Test in both study areas, rates in young children were similar with both tests in both areas.

**Conclusions:**

These results extend the previously reported superior sensitivity of the FTS to areas with low residual infection rates following MDA, and this could affect mapping and post-MDA survey results in adults. However, our findings suggest that results of transmission assessment surveys (TAS) performed in school-aged children are likely to be similar with both tests.

## Background

Lymphatic filariasis (LF) is a major neglected tropical disease (NTD) that the World Health Organization (WHO) has targeted for elimination by the year 2020 [1]. The Global Programme to Eliminate Lymphatic Filariasis (GPELF) recommends repeated rounds of mass drug administration (MDA) with antifilarial medications to interrupt transmission of the nematode worms that cause LF. Almost 5 billion treatment doses of these drugs were distributed in 60 disease-endemic countries between the years 2000 and 2012 [2]. WHO guidelines rely heavily on diagnostic test results to manage different phases of LF elimination programs. Prior studies have shown that tests that detect circulating filarial antigen-(CFA) are much more convenient and more sensitive for detecting *Wuchereria bancrofti* infections than the traditional method of detecting microfilariae (Mf) in night blood samples by microscopy [3, 4]. For this reason, WHO guidelines recommend CFA tests as a primary tool for mapping the distribution of the infection. In addition, CFA tests are used in transmission assessment surveys (TAS) that systematically sample young school-aged children to detect recent infections [5, 6]. TAS results figure prominently in surveys based on WHO guidelines for stopping MDA and (later) for verification of elimination [7–9].

The first field-friendly test for CFA (the ICT Filariasis Test, produced by ICT Diagnostics, Balgowah, NSW, Australia) was marketed in 1997 [10]. This test was transferred to Binax, Inc. (Portland, ME) in 2000, and as the Binax Now Filariasis card test (Card Test), it has been widely used by LF elimination programs for mapping and post-MDA surveillance. Alere Scarborough (a successor company to Binax) has recently introduced a next generation CFA test called the Alere Filariasis Test Strip (FTS). This immunochromatography test uses the same reagents as the Card Test on a different platform. The FTS is less expensive than the Card Test, and it has a much longer shelf life. A laboratory evaluation showed that the FTS has a lower limit of detection than the Card Test, and it detected 27 % more positive results than the Card Test in a field study that was performed in a high endemicity setting in Liberia [11]. Despite these advantages, more information is needed from different settings before the new test can be endorsed as a substitute for the Card Test. In particular, some experts have expressed concern that a more sensitive CFA test might significantly raise the bar for elimination if it detected many more infections in children than the Card Test during TAS. Therefore, the purpose of the present study was to compare the performance of the FTS and the Card Test in two areas in Asia that have low residual *W. bancrofti* infection rates and infection intensities following multiple rounds of MDA with diethylcarbamazine plus albendazole. The studies were performed in densely populated semi-urban areas where *W. bancrofti* is transmitted by *C. quinquefasciatus*.

## Methods

### Antigen detection tests and protocol

BinaxNOW® Filariasis card tests were purchased from Alere Scarborough, Inc., Scarborough, ME, USA. Alere Filariasis Test Strips (FTS) from the same manufacturer were provided for this study by the company at no cost. Site specific study protocols and standard operating procedures for the study were developed by Washington University investigators in consultation with collaborating scientists in Sri Lanka and Indonesia.

### Ethical approval

The human studies protocols were approved by an ethical review committee at the University of Ruhuna (Galle, Sri Lanka), and by institutional review boards at the University of Indonesia, and Washington University School of Medicine. All adult participants in the study provided written informed consent. Participation of minors required their assent and written consent from one parent.

### Field studies using the filarial antigen tests

#### Sri Lanka: study areas and subjects

The studies were performed in two Grama Niladhari (GN) divisions, Matotagama in Matara district and Unawatuna-West in Galle district. Matotagama received 12 semiannual rounds of mass drug administration for LF with diethylcarbamazine (DEC) plus albendazole in 2002–2006, while Unawatuna received 5 rounds of MDA during that period [12, 13]. These GN divisions were chosen for this study, because post-MDA surveillance studies documented low-level persistence of LF [8, 13]. Household maps were prepared for each GN division by project personnel prior to the surveys. The total registered populations were 1,561 in Matotagama and 745 in Unawatuna-West. Previous post-MDA surveys (ages ≥ 2) in these two GN divisions found CFA rates by Card Test of 3.7 % in Unawatuna West and 4 % in Matotagama.

The surveys were conducted in January-April 2014 by three blood collection teams comprised of one medical officer (team leader/phlebotomist), one field assistant, and two test readers. CFA tests were performed in the field at the time of blood collection. The study surveyed 5 neighborhood clusters in each GN that were selected based on ease of access and results of past LF surveys. All households in these clusters were visited, and persons over 5 years of age were invited to participate in the study. The mean number of persons tested per household was 3.1, SD = 1.4 (range 1–9). All persons with positive CFA tests were treated with a single oral dose of DEC 6 mg/kg plus albendazole 400 mg. Persons with microfilaremia also received additional DEC to complete a 14 day course of treatment.

#### Indonesia: study area and subjects

This study was performed in the municipality of Pekalongan, a city on the northern coast of central Java that is endemic for bancroftian filariasis. Pekalongan’s 282,000 residents live in 47 villages/neighborhoods that are served by 12 primary health centers (Puskesmas; http://www.dinkesjatengprov.go.id/). The village of Kertoharjo that was chosen for this study received 4 rounds of MDA with diethylcarbamazine plus albendazole between 2011 and 2014. A survey in Kertoharjo in 2012 (12 months after the first round of MDA) found Mf (by 60 μl thick smear) and CFA rates by Card Test of 2.2 and 4.1 %, respectively. The fourth round of MDA was distributed in Kertoharjo in the summer of 2014, approximately 2 months prior to the current study, which was performed in August-September 2014. There were two components to the Kertoharjo study. A community survey tested residents > 5 years of age in a convenience sample of households. The second part of the Kertoharjo study tested children ages 6 to 15 who attended schools in the village. The school survey was conducted to increase the number of school-aged children in the study. This is because many parents were reluctant to wake children for blood collection during the community survey, which was performed in the evening. As in Sri Lanka, all persons with positive CFA tests were treated with a single oral dose of (DEC) 6 mg/kg plus albendazole 400 mg.

### Blood collection, antigen and microfilaria testing

Washington University investigators trained staff in Sri Lanka and Indonesia on standard operating procedures for blood collection and antigen testing. Training in Sri Lanka included laboratory testing of approximately 200 samples collected during a pilot study in Hill Side Watta in Galle district. Blood samples for this pilot study were collected in Ethylenediaminetetraacetic acid (EDTA) coated blood collection tubes (Fisher Scientific, Pittsburgh, PA), and the antigen tests were performed in a laboratory the next morning. Results from this pilot survey showed a much greater discordance between the tests than expected, with more positives by FTS. Most people with discordant test results with EDTA blood were negative by both tests when they were retested in the field with finger prick blood according to the manufacturer’s instructions, and this method was used for the surveys in Unawatuna West, Matatogama, and Pekalongan that are reported in this paper. The Indonesian study was initiated without a pilot field study.

Blood was collected with sterile, single use, contact activated BD-microtainer lancets (Fisher Scientific). Community blood samples were collected between 16:00–23:00 h in Sri Lanka and between 10:00–22:00 h in Indonesia. Barcode stickers were used to link CFA test results with participants’ demographic records. Barcode stickers were attached to FTS and Card tests, and the stickers were used to secure FTS in their plastic boats. Blood samples were applied to the two CFA tests according to the manufacturer’s instructions. Test Strips and Card Tests were read by different technicians. Test results were recorded independently at 10 min, and results were verified by a third reader who served as a supervisor. Test results were scored according to the intensity of the test “T” line relative to that of control “C” line as previously described [11]. Briefly, scores for both tests were recorded as follows: 0, no T line visible; 1, T line is weaker than the C line; 2, T line is equal to the C line; 3, T line is stronger than the C line. The Sri Lanka study also scored the tests at 30 min and at 12 h.

Persons with positive antigen tests at 10 min had blood collected at night for Mf testing. Finger prick blood samples were collected between the hours of 20:00 and 23:00 in Indonesia and between 21:00 and 23:00 in Sri Lanka. Three-line thick blood smears (a total of 60 μl of blood per smear) were stained with Giemsa and examined for Mf by microscopy. The Sri Lanka study also used membrane filtration of 1 ml of venous blood for Mf detection [14].

### Data collection, management, and analysis

Demographic information (name, household, age, gender, and documentation of informed consent) was recorded on preprinted paper forms. Identifying data, specimen ID, FTS, and Card test results were linked using barcode stickers, and results were entered into Microsoft Excel files. Unique identifiers were removed from data files prior to analysis. The Sri Lanka study also collected household GPS coordinates with personal digital assistants (PDA) (HP iPAQ 211, Hewlett Packard, Palo Alto, CA). Locations were plotted using ArcGIS 10.2.1 (ESRI, Redlands, CA), and waypoints were color coded to mark households with one or more positive filarial antigen tests.

The software packages PASW Statistics 18 (SPSS, IBM Corporation, Armonk, NY) and R 3.1.1. [15] were used for data analysis. The Chi-square test was used to compare results obtained with the two antigen tests. This analysis and the calculation of % agreement between the two antigen tests only considered samples with valid results for both tests. The Effect of age on CFA test results was also assessed using a generalized linear model (GLM) with a binomial family function that also considered gender [15]. Age was logx transformed for the Indonesia data to meet assumptions for normality. This was not required for the Sri Lanka data. The Wilcoxon signed rank test was used to assess the significance of differences in test scores obtained by FTS and Card Test.

## Results and discussion

### Results of CFA comparison studies in Sri Lanka and Indonesia

Eight hundred fifty-five subjects (Age range 5–91, median 35) from Unawatuna west and Matotagama, Sri Lanka and 815 subjects (Age > 5–77, median 11) in Pekalongan, Indonesia were enrolled in the test comparison studies. 491 of 815 (60 %) Indonesian study subjects were school-aged children (Age 6–15). 43 % of enrolled subjects were males in both countries.

Test results are summarized in Table 1. 852 subjects in Sri Lanka and 782 subjects in Indonesia had valid results with both CFA tests, and these results were used for the comparative analysis. There were very few invalid tests in the Sri Lanka study (1 Card Test and 2 FTS). In contrast, there were 4 invalid Card Tests and 33 (4.2 % of the total) invalid FTS results in Indonesia. Invalid FTS results in Indonesia were due to blood clotting in the blood collection device or to too little blood collected or applied to the Test Strip, both of which would decrease flow of serum on the Test Strips. Most of the invalid FTS results occurred in the first few days of the study, and this problem decreased after the technicians gained experience with the plastic blood collection devices. Besides causing invalid test results, partial clotting or incomplete flow could also reduce the sensitivity of the FTS by reducing the volume of serum (FTS) or plasma (Card Test) that flows across the “T” line. The pilot study in Sri Lanka provided field training with both tests, and this may account for the low rate of invalid FTS and the lack of Card Test-positive/FTS-negative results in the Sri Lanka study.

**Table 1:** Cross-tabulation of filarial antigen test results obtained by the Card Test and the Test Strip

Country	No. tested				
			Card Test result		
		FTS result	Positive	Negative	Totals
Sri Lanka	852	Positive	18	25	43
		Negative	0	809	809
		Totals	18	834	852
		% Agreement; Kappa score (95 % CI): 97.07 %; 0.578 (0.430–0.725). Chi-square = *P* <0.0001			
Indonesia	778	Positive	38	12	50
		Negative	3	725	728
		Totals	41	737	778
		% Agreement; Kappa score (95 % CI): 98 %; 0.825 (0.739–0.912). Chi-square = *P* <0.0001			

Results shown are for 852 and 778 blood samples from Sri Lanka and Indonesia with valid results with both filarial antigen tests

*FTS* Filariasis Test Strip

Study personnel in both countries mentioned the problem of securing the light weight FTS in plastic holders, and they complained that it is more difficult to label the thin plastic FTS compared to the Card Test. As mentioned above, there is also a learning curve for use of the plastic micropipettes that come with the FTS kits. These factors make the FTS slightly more difficult to use than the Card Test.

**Fig. 1:**
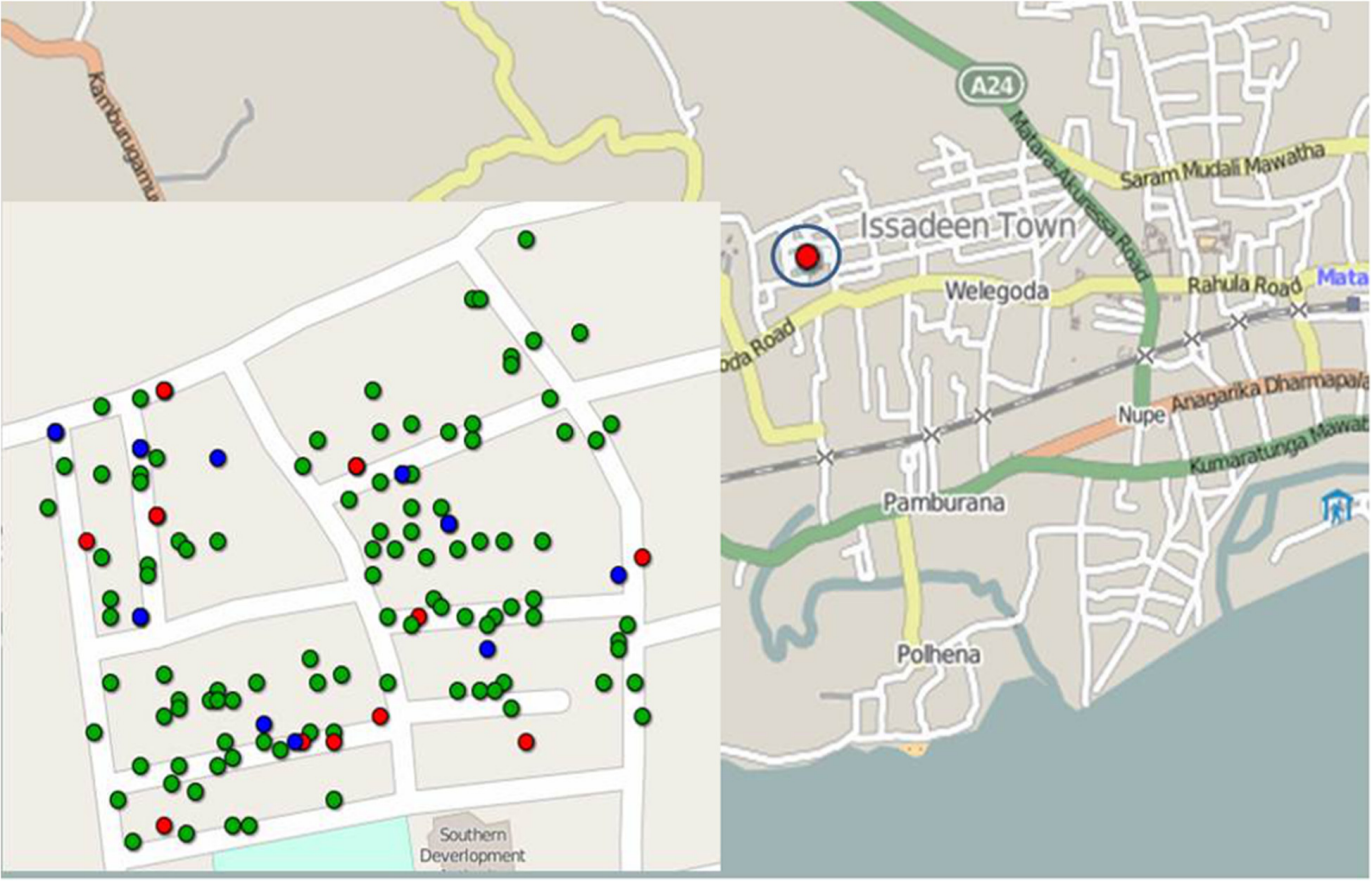
The background map shows the location of Matotagama village (circle) in Matara district, Sri Lanka. The inset map on the left shows households included in the survey. Red dots indicate households with one or more residents with positive Filariasis Test Strip (FTS) results where all residents had negative Card Tests. Blue dots show households with residents who were positive for circulating filarial antigenemia (CFA) by FTS and the Card Test. Green dots show houses with no positive CFA test results. All persons in this study site with positive Card Test results were also positive by FTS

While almost all samples that were positive by Card Test were also positive by FTS, many samples that were negative by Card Test were positive by FTS. This finding is illustrated in Fig. 1, which compares the distribution of houses with residents with positive CFA tests by FTS and Card Test. The FTS/Card Test ratio (the number of positive FTS divided by the number of positive Card Tests for people with valid results for both tests) was 2.33 in the Sri Lanka study but only 1.22 in Indonesia. Exclusion of results for 3 samples with positive Card Test and negative FTS results raises the Indonesian FTS/Card Test ratio to 1.32.

### Associations of age and gender with CFA test results

CFA rates differed by age and gender in the Sri Lanka study. Antigen prevalence rates tended to increase with age, and this trend was stronger for FTS results (Table 2). Only 1 of 53 children less than 11 years of age (most of them born after Sri Lanka’s MDA program was completed in 2006) had a positive CFA test result (by FTS only). CFA rates were also higher in males than in females by FTS in Sri Lanka (31/359 or 8.7 % *vs.* 12/493 or 2.4 %, *P* < 0.05). The CFA rate by FTS was higher in adults than in children (5.7 % in people with age > 15 *vs.* 1.5 % in those with ages 6–15, *P* <0.05). The same age and gender trends were observed in the Indonesia study: CFA rates by FTS were higher in males than in females (31/332 or 9.3 % *vs*. 19/446 or 4.3 %, *P* <0.05), and rates in adults (age > 15) were higher than rates in children (10 % *vs*. 4 %, respectively, *P* <0.05). Higher CFA rates in males in both study sites may be due to higher baseline infection rates in males prior to MDA or to lower MDA compliance in males. CFA rates in children < 11 years of age were similar by both tests in the Indonesia study (3.5 % by FTS and 3.2 % by Card Test). This result suggests that a school-based transmission assessment survey in this area would have failed by FTS or by Card Test.

**Table 2:** Circulating filarial antigenemia test results by age as assessed in Sri Lanka and Indonesia

	Sri Lanka				Indonesia			
Age (years)	Test Strip		Card Test		Test Strip		Card Test	
	POS / total	% (95 CI)	POS / total	% (95 CI)	POS / total	% (95 CI)	POS / total	% (95 CI)
<11	1/53	1.9 (0.3–9.9)	0/53	0.0 (0–6.9)	12/353	3.4 (1.9–5.8)	11/353	3.1 (1.7–5.4)
11–20	4/172	2.3 (0.9–5.8)	0/172	0.0 (0–2.2)	8/126	6.3 (3.2–12.0)	5/126	4.0 (1.7–8.9)
21–30	6/125	4.8 (2.2–10.1)	4/125	3.2 (1.3–7.9)	10/75	13.3 (7.4–22.8)	8/75	10.7 (5.5–19.6)
31–40	9/154	5.8 (3.1–10.7)	4/154	2.6 (1.0–6.5)	9/88	10.2 (5.4–18.3)	7/88	8.0 (3.9–15.5)
41–50	5/142	3.5 (1.5–8.0)	2/142	1.4 (0.4–5.0)	2/70	2.9 (0.8–9.83)	2/70	2.9 (0.8–9.83)
51–60	8/113	7.1 (3.6–13.4)	4/113	3.5 (1.4–8.8)	8/53	15.1 (7.8–27.5)	7/53	13.2 (6.5–24.8)
≥61	10/93	10.8 (5.9–18.7)	4/93	4.3 (1.7–10.5)	1/13	7.7 (1.4–33.3)	1/13	7.7 (1.4–33.3)
Total	43/852	5.0 (3.7–6.7)	18/852	2.1 (1.3–3.3)	50/778	6.4 (4.9–8.3)	41/778	5.3 (3.9–7.0)

We also tested the effect of age on antigenemia rates using a GLM that controlled for the effect of gender. Higher age was significantly associated with positive CFA test results for both tests in both the Sri Lankan and in Indonesian study sites with *P* values that were no higher than < 0.02 for all four comparisons.

### Comparison of card test and FTS scores

FTS scores (based on the intensity of “T” lines) tended to be higher than Card Test scores (Table 3). In the Sri Lanka survey, the mean (SD) test scores for samples with positive FTS or Card Test were 1.72 (0.79) and 0.67 (0.96), respectively (*P* < 0.0001). In Indonesia, the mean (SD) scores for samples with positive FTS or Card Test were 1.64 (0.87) *vs*. 0.98 (0.72), respectively (*P* <0.0001). When combined data from both countries is considered, most subjects with positive FTS and negative Card Test results had FTS scores of 1 (30 of 37 or 81 %), and only 15 of 45 (33 %) samples with FTS scores of 1 had positive Card Tests. On the other hand, 41 of 48 (85 %) of samples with scores of 2 or 3 by FTS had positive Card Test results.

**Table 3:** Cross-tabulation of test scores^a^ obtained with the Test Strip and Card Test in Sri Lanka and Indonesia

		Sri Lanka					Indonesia				
		Card Test score					Card Test score				
		0	1	2	3	Total	0	1	2	3	Total
Test Strip Score	0	809	0	0	0	809	725	3	0	0	728
	1	20	1	0	0	21	10	14	0	0	24
	2	5	8	0	0	13	2	13	0	0	15
	3	0	2	3	4	9	0	3	7	1	11
	Total	834	11	3	4	852	737	33	7	1	778

^a^Test results were scored based on the intensity of the ‘T’ line at 10 min as described in Methods. Results shown are for samples that were positive by either test

### CFA test result stability

Stability of CFA test results was assessed in the Sri Lanka study. Tests that were negative at 10 min were read again at 30 min and 12 h. More FTS turned positive at both time points compared to Card Tests (1.7 % vs. 0.5 % at 30 min, and 3.8 % vs. 1.4 % at 12 h, respectively). These results provide an indication of what to expect if there is a delay in reading these CFA tests. While it is possible that some or all of the delayed positive results were true positives, both tests should be read at 10 min as recommended by the manufacturer.

### Microfilaremia test results

Night blood testing for Mf was performed for most of the study participants who had positive CFA results by either the Card Test or FTS. Six of 43 (14 %) Sri Lankan subjects with positive FTS results had microfilaremia (range 1–1245 Mf; mean ± SD = 276 ± 485 per ml) by membrane filtration. All six of these subjects had FTS scores ≥ 2. However, only 4 of 43 (9.3 %) of people with positive FTS results had Mf by thick smear (range 2–45 Mf; mean ± SD = 13 ± 19 per 60 μl). Four of 18 (22 %) people with positive Card Tests had Mf by both membrane filtration and by thick smear. While all four people with Mf by thick smear had positive Card Test and FTS results, only one of two people with Mf by filter but not by thick smear had a positive Card Test.

In the Indonesia study, 8 of 34 (23.5 %) participants with positive FTS results were Mf positive by night blood smear (range 2–52 Mf per 60 μl; mean ± SD = 5 ± 13). In contrast, a higher proportion of people with positive Card Tests were Mf positive (8 of 25, 32 %). All of the Mf positive subjects were positive for CFA by both the FTS and Card Test, but only 5 of 8 had FTS scores ≥ 2. Membrane filtration was not performed in the Indonesia study. These results show that only a minority of people with positive CFA tests have microfilaremia and that Mf rates for those with positive Card Tests tend to be higher than rates in those with positive FTS results.

### Additional discussion points

This study has provided useful information on the performance of the FTS and the Card Test in areas with low residual LF infection rates and infection intensities following multiple rounds of MDA. The finding that CFA rates were higher by FTS than by Card test is consistent with the known superior analytical sensitivity of the FTS. We do not know why the difference in CFA rates by FTS and Test Strip positivity was so much higher in Sri Lanka than in Indonesia. It is interesting that this difference was much more striking in adults than in children.

This study has also confirmed the value of semi-quantitative scoring for FTS and Card Test results. FTS scores were significantly higher than Card Test scores in both study sites. In addition, combined data from both study sites showed that most people with microfilaremia (11 of 14 or 79 %) had FTS scores ≥ 2.

The improved sensitivity of the FTS increases its value for mapping LF endemicity, as illustrated in Fig. 1. The FTS will also be superior to the Card Test for post-MDA surveys of adults and for studies that assess the macrofilaricidal effects of new treatments. Since CFA rates by both tests were similar in children less than 11 years of age in both study sites, a switch from the Card Test to the FTS should not dramatically affect post-MDA transmission assessment survey (TAS) results, since those surveys sample young school aged children.

## Conclusions

Results from this study support the idea of using the FTS instead of the Card Test in LF elimination programs. The smaller blood requirement (75 μl), lower cost, and longer shelf life of the FTS also favor the switch. It would be helpful if the FTS kits could be modified to make them more user-friendly. As with any new point of care test, technicians should be provided with training and practical experience performing the test before it is deployed in national surveys. Special training materials might help to ease the transition from the Card Test to the FTS.

## Abbreviations

Card Test: BinaxNOW Filariasis card test
CFA: Circulating filarial antigen
DEC: Diethylcarbamazine
EDTA: Ethylenediaminetetraacetic acid
FTS: Filariasis Test Strip
GLM: Generalized Linear Model
GN: Grama Niladhari
GPELF: Global Programme to Eliminate Lymphatic Filariasis
GPS: Global Positioning System
LF: Lymphatic Filariasis
MDA: Mass Drug Administration
Mf: Microfilaria
NTD: Neglected Tropical Diseases
TAS: Transmission Assessment Survey
WHO: World Health Organization

## References

[1] WHO (2010). Lymphatic Filariasis. Progress Report 2000–2009 and Strategic Plan 2010–2020. WHO/HTM/NTD/PCT/20106).

[2] WHO (2013). Global programme to eliminate lymphatic filariasis: progress report. Wkly Epidemiol Rec / Health Section Secretariat League Nations 2014.

[3] Weil GJ, Ramzy RM (2007). Diagnostic tools for filariasis elimination programs. Trends Parasitol.

[4] Gass K, de Rochars MV B, Boakye D, Bradley M, Fischer PU, Gyapong J, Itoh M (2012). A multicenter evaluation of diagnostic tools to define endpoints for programs to eliminate bancroftian filariasis. PLoS Negl Trop Dis.

[5] WHO (2000). Preparing and implementing a national plan to eliminate lymphatic filariasis (in countries where onchocerciasis is not co-endemic).

[6] WHO (2005). Monitoring and epidemiological assessment of the programme to eliminate lymphatic filariasis at implementation unit level WHO/CDS/CPE/CEE/200550.

[7] WHO (2011). Monitoring and epidemiological assessment of mass drug administration in the Global Programme to Eliminate Lymphatic Filariasis: A manual for national elimination programmes. . WHO, (WHO/HTM/NTD/PCT/2011 4).

[8] Rao RU, Nagodavithana KC, Samarasekera SD, Wijegunawardana AD, Premakumara WD, Perera SN (2014). A comprehensive assessment of lymphatic filariasis in Sri Lanka six years after cessation of mass drug administration. PLoS Negl Trop Dis.

[9] Rebollo MP, Mohammed KA, Thomas B, Ame S, Ali SM, Cano J (2015). Cessation of mass drug administration for lymphatic filariasis in Zanzibar in 2006: was transmission interrupted?. PLoS Negl Trop Dis.

[10] Weil GJ, Lammie PJ, Weiss N (1997). The ICT Filariasis Test: A rapid-format antigen test for diagnosis of bancroftian filariasis. Parasitol Today.

[11] Weil GJ, Curtis KC, Fakoli L, Fischer K, Gankpala L, Lammie PJ (2013). Laboratory and field evaluation of a new rapid test for detecting *Wuchereria bancrofti* antigen in human blood. AmJTrop Med Hyg.

[12] WHO. Neglected tropical diseases. PCT data bank [http://www.who.int/neglected_diseases/preventive_chemotherapy/lf/en/].

[13] Yahathugoda TC, Weerasooriya M, Samarawickrema WA (2013). An independent evaluation of the programme for the elimination of lymphatic filariasis. Galle Medical Journal.

[14] Desowitz RS, Hitchcock JC (1974). Hyperendemic bancroftian filariasis in the Kingdom of Tonga: the application of the membrane filter concentration technique to an age-stratified blood survey. AmJTrop Med Hyg.

[15] R Core Team. A Language and Environment for Statistical Computing. R Foundation for Statistical Computing, Vienna, Austria. 2014.

